# A Novel Quantitative Analysis in Myocardial Perfusion Imaging: How does it compare to the “Golden Eye”?^☆^

**DOI:** 10.1016/j.zemedi.2026.03.009

**Published:** 2026-03-21

**Authors:** Yue Guo, Juncheng Ni, Esteban Lucas Solari, Alberto Villagran Asiares, Stephan G. Nekolla

**Affiliations:** a Nuklearmedizinische Klinik und Poliklinik, TUM Klinikum, Klinikum rechts der Isar, School of Medicine and Health, Technical University of Munich, Munich, Germany; b Deutsches Zentrum für Herzinsuffizienz, Universitätsklinikum Würzburg, Würzburg, Germany; c Deutsches Zentrum für Herz-Kreislauf-Forschung eV, partner site Munich Heart Alliance, Munich, Germany; d Klinik und Poliklinik für Kinder- und Jugendpsychiatrie, Psychosomatik und Psychotherapie. Klinikum der Ludwig-Maximilians-Universität München, Munich, Germany

**Keywords:** Cardiac imaging, Myocardialperfusionimaging, Nuclearcardiology, SPECT, Deep learning

## Abstract

Myocardial Perfusion Imaging is widely used to evaluate left ventricular perfusion in patients with ischemic heart disease. Semi-quantitative scores, such as Total Perfusion Deficit (TPD), are frequently used for the assessment of extent and severity of disease. TPD scores are based on a frequentist analysis of tracer distribution in perfusion polar maps, which are 2D polar representations of radiopharmaceutical tracer uptake in the left ventricular cardiac wall. In this paper, we propose a novel quantification method for MPI examinations. Our method approaches the problem from an inverse perspective to TPD, allowing the assessment of the severity and extension of abnormal perfusion from the synergetic contribution of all the left ventricular tracer uptake in a polar map. We analytically demonstrate that TPD can be viewed as a specific instance of a broader approach. Furthermore, we explore how to establish the quantitative analysis in a general approach. To test our new method, we leveraged a standard deep learning architecture for the prediction of abnormal perfusion and used gradient-based class activation maps as a measure of the abnormal perfusion territory, which facilitated the quantification of both the severity and extent of the perfusion deficit. In the absence of ground-truth data such as survival rates or major adverse cardiovascular events (MACE) criteria, our results demonstrate both statistical and visual consistency with the conventional TPD.

## Introduction

Cardiovascular diseases (CVD) are the leading cause of death worldwide. Myocardial Perfusion Imaging (MPI) using radiopharmaceuticals and single-photon emission computed tomography (SPECT) imaging is the standard noninvasive cardiac imaging technique to evaluate myocardial ischemia and its associated functional damage to the heart during coronary artery disease (CAD). During an examination, stress and rest image acquisitions are performed sequentially, generating matching perfusion polar maps under both conditions.

Quantification of myocardial perfusion images improves the reliability and reproducibility of clinical interpretation [1] and is widely used in clinical settings for diagnosis and prognosis of suspected CAD [2]. The location and severity of perfusion defect can be visually evaluated using the American Heart Association (AHA) 17-segment model [3]. Semi-quantitative analysis is a well-established method for interpreting nuclear cardiology studies in a highly standardized way, as described in the guidelines from the American Society of Nuclear Cardiology (ASNC). It offers robust diagnostic and prognostic value [4]. The use of sophisticated software for these tasks belongs to the very early applications in nuclear medicine using conventional statistical methods [5] as well as neural networks [6].

The current clinical paradigm of quantitative analysis of perfusion polar maps is based on Total Perfusion Deficit (TPD) [7], which is a numerical score representing the percentage extent and severity of abnormal myocardial perfusion with respect to a specific normal database. The normal limits are defined for each specific cardiac segment in an N-segment model (typically, N=17) of the left ventricle (LV) with respect to the mean uptake of a CAD-negative patient sample [8], [9], [10]. However, this approach has been reported to have limitations, as it assumes a normal distribution of the per-segment uptakes within the negatively diagnosed population [11]. Additionally, it assumes the independent behavior of different perfusion segments, even though these segments belong to the same perfusion territory.

The aim of quantification of MPI can be divided in three categories: localization of the abnormal region, evaluation of the extent and severity of the disease and classification of normal/abnormal polar maps. Most of the attention in previous studies was focused on improving classification results [12], [13], [14], [15], [16], [17]. Progress has also been made regarding the clinically-relevant localization of the hypo-perfused region [18], [19], including explainable deep learning models to visually assess obstructive CAD regions, and region-based classification [12], [20], [21], [22]. However, their results are predominantly based on the restricted per-vessel 3-segment model of the main coronary vessels’ perfusion territory (LAD, LCX, RCA) [18]. A more useful localization would consider at least the 17-segment model.

The aim of this paper is to propose a novel quantification system to tackle the intrinsic problems of the TPD approach to evaluate the segment-wise severity and extent of the perfusion abnormality using a standard deep learning architecture. Additionally, we present the theoretical framework which offer insights for future quantitative system design in nuclear cardiology.

## Material and methods

Clinical Reference Standard: Total Perfusion Deficit

**Fig. 1:**
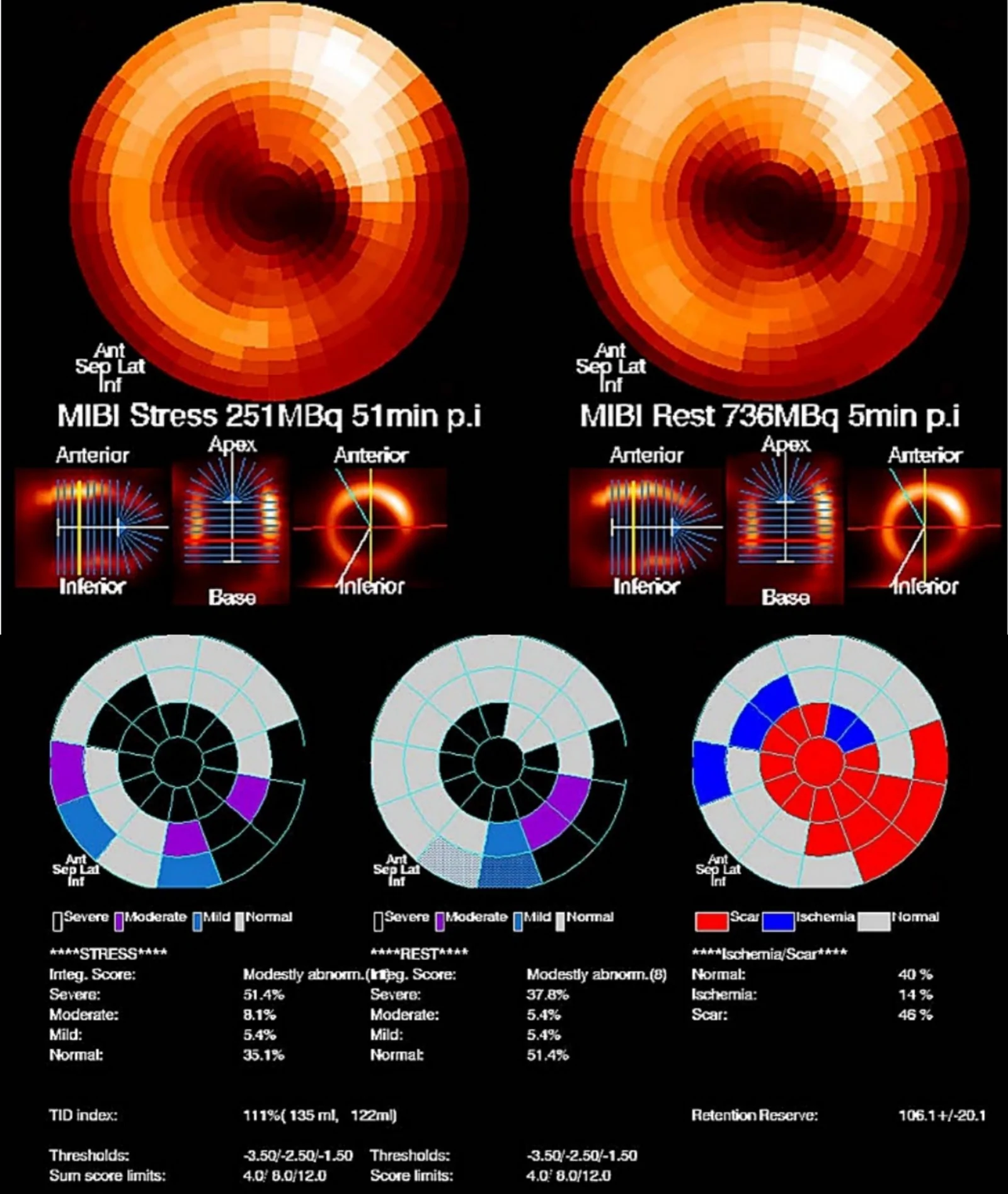
TPD analysis using software MunichHeart. Above: stress (left) and rest (right) perfusion polar maps, including their original LV SPECT images. Below: per-segment deficit severity score (0-3) and extent (%) for stress (left) and rest (middle) polar maps (black/purple/blue/gray: severe (3) / moderate (2) / mild (1) / normal (0) region). Right: according ischemia and scar segmentation (blue/red).

To perform TPD quantification, the 3D LV perfusion distribution is mapped into 2D polar maps to standardize wall sizes and shapes. The uptake in each segment $x_{\mathrm{ij}}$ is compared to a gender-specific normal uptake distribution database derived from a healthy population. Quantitative analysis was performed using the commercial software *MunichHeart* [23], which serves as the clinical reference standard for this study (Fig. 1).

Instead of a simple thresholding, TPD utilizes a continuous statistical scoring system. A standardized z-score $z_{\mathrm{ij}}=-\frac{x_{\mathrm{ij}}-\mu_{i}}{\sigma_{i}}$ is calculated for each segment, where $\mu_{i}$ and $\sigma_{i}$ are the mean and standard deviation of the normal database. Segments with uptake falling significantly below the mean (typically below 2.5σ) are assigned a severity score ranging from 0 (normal) to 3 (severely abnormal).

Global deficit scores, including Summed Stress Score (SSS) and Summed Rest Score (SRS), are calculated as the sum of individual segment scores. To account for the varying size of defects, a total severity score $s_{j}$ is defined as the sum of the fractional extent $f_{k}$ of the abnormal regions multiplied by their severity scores *k*:

(1) $$s_{j}=\sum_{k=0}^{3}k*f_{k}\propto TPD.$$

This semi-quantitative scoring provided the segment-level and patient-level labels used to train and validate our deep learning approach.

From TPD to Deep Learning: A Generalized Framework

**Fig. 2:**
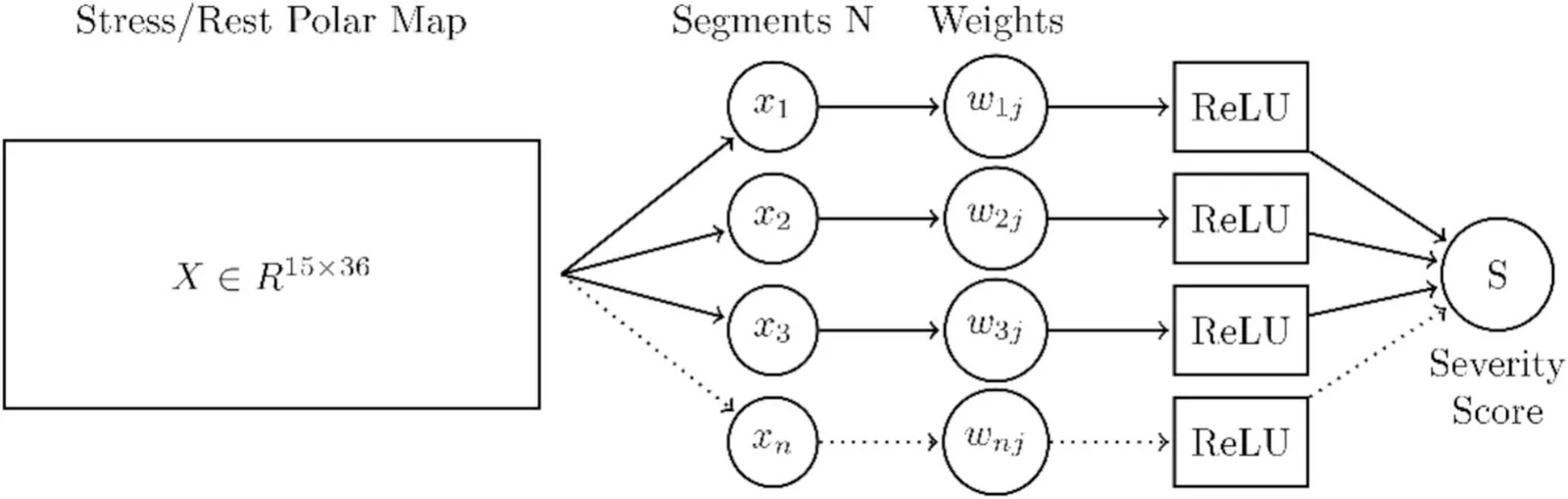
The TPD perceptron. A polar map is first transformed into *N* segments through average pooling. The continuous severity score is then obtained through the weighted sum of all $x_{\mathrm{ij}}$ segments with bias (non-trainable affine layer) and a ReLU activation (corresponding to the max operator).

To bridge the gap between traditional statistical methods and modern data-driven approaches, we analyzed the mathematical structure of the conventional TPD scoring system. We demonstrate that the conventional TPD score can be mathematically formulated as a specific, linear instance of a broader quantification function. As derived in detail in Appendix A, the severity score $s_{j}$ of segment $x_{\mathrm{ij}}$ in TPD can be expressed as:

(2) $$s_{j}=\sum_{i=1}^{N}\left(\frac{\mu_{i}-x_{\mathrm{ij}}}{\sigma_{i}}-T\right),$$

where the weights are determined by the mean $\mu_{i}$ and inverse standard deviation $\sigma_{i}$ of the normal database, and the thresholding (T) logic functions as a Rectified Linear Unit (ReLU) activation. Structurally, this standard TPD equation is equivalent to a linear regression model with a ReLU activation function (Fig. 2), analogous to a single-layer perceptron. This implies that the current clinical standard is effectively a specific, linear instance of a broader neural network framework.

However, this linear formulation relies on two strong assumptions: the independence of individual segments and the linear relationship between tracer uptake and perfusion [24]. These assumptions may not hold true for complex physiological conditions or all tracers. While polynomial fitting methods (such as GMDH) could theoretically extend this framework to non-linear cases, they suffer from exponential computational complexity as the number of interaction terms increases (Appendix B).

To overcome this, we generalize the function to a non-linear, voxel-wise deep learning model:

(3) $$s_{j}=f_{\theta }\left(X_{j}\right),$$

where $X_{j}$ is the high-resolution input map and θ represents the learnable parameters of a Convolutional Neural Network (CNN). Equation $\left(3\right)$ aligns with our intuitive understanding that the human heart is better represented along a continuum of severity rather than as a binary phenomenon [25]. This approach allows the quantification system to learn complex, non-linear spatial dependencies directly from the data, overcoming the intrinsic limitations of the linear TPD model while maintaining a theoretical connection to the established clinical standard.

Image Acquisition and Diagnosis

We retrospectively analyzed 1028 perfusion SPECT studies acquired on a digital, dedicated cardiac SPECT system (D-SPECT, Spectrum Dynamics, Haifa, Israel) between 2022 and 2023 from TUM Klinikum Rechts der Isar, Munich. In the one-day protocol, ^99^*^m^*Tc – Sestamibi was injected to each patient twice: first during the stress acquisition, and after a 1-hour waiting period, 3 times as much radiotracer during the rest acquisition to account for the fact that the tracer binds rather irreversibly and the half-life is about 6 hours. Stress and rest polar maps were generated and analyzed using the nuclear cardiology analysis software MunichHeart which extracts the uptake data from the 3D data volume [26] to finally assess conventional TPD. All perfusion studies have been evaluated by experts and normal diagnosis criteria were defined according to the clinical routine, as the combination of:

1) normal perfusion extent larger than 91%;
2) transient ischemia dilation (TID, the ratio of the stress to rest LV volume) smaller than 120%;
3) endocardial volume under stress and rest condition smaller than 120 cm^3^.

Data Preprocessing

For each study we obtained four 15×36 arrays using the internal MunichHeart polar map data format: stress intensity, rest intensity, and the corresponding voxel-volume maps for stress and rest (derived from the polar geometry). The volume information was retained for computing the fractional extent of abnormal tissue in the post-processing step, but it was not used as a separate input channel to the CNN. To ensure the model learned directly from raw data, the final network inputs consisted of normalized pixel intensities rather than derived features. Following the standard *MunichHeart* protocol, these intensities were normalized relative to the mean value of the maximum segment among the six sectors.

For the CNN input, the stress and rest polar maps were treated as two separate images. Each 15×36 intensity map (stress or rest) was linearly resized to 224×224 using bilinear interpolation. Because the ResNet-18 backbone expects three input channels, the resulting single-channel image was replicated across the three channels. Therefore, each stress or rest polar map was therefore represented as a 3×224×224 tensor. No additional information was encoded in the extra channels; they are identical copies of the normalized intensity image and serve purely to match the input format of the pre-trained without requiring modification to the initial convolutional layers.

Some authors have preferred to add the stress and rest images together as a unique input [27]. Given that each polar map contributes different information, and that we later need the localization in each of the images to differentiate between ischemic and scarred tissue, therefore the stress and rest images are treated as separate inputs. Other authors have previously trained two separate models for the localization of abnormalities in stress and rest polar maps. As shown in Fig. 2 [28], this approach sometimes results in completely mismatched localizations for the stress and rest images, which do not correspond to the clinical reality. We consider it preferable to train and infer with a same single model $f_{\theta }$ using both rectangular maps individually in order to refrain from the mismatched problem. This is similar to the traditional TPD implementation, which uses the same statistical model (*θ: {µ_i_, σ_i_})* for both stress and rest polar maps.

Our approach encodes the severity of the abnormal perfusion into a voxel-wise 4-point scale ($s_{0}-s_{3}$). The per-image severity $s_{j}$ will then be computed as the sum of the fractional extent of the abnormal regions $r_{k}$ multiplied by their semi-quantitative severity scores as the training target.

Deep Learning Pipeline and Model Selection

**Fig. 3:**
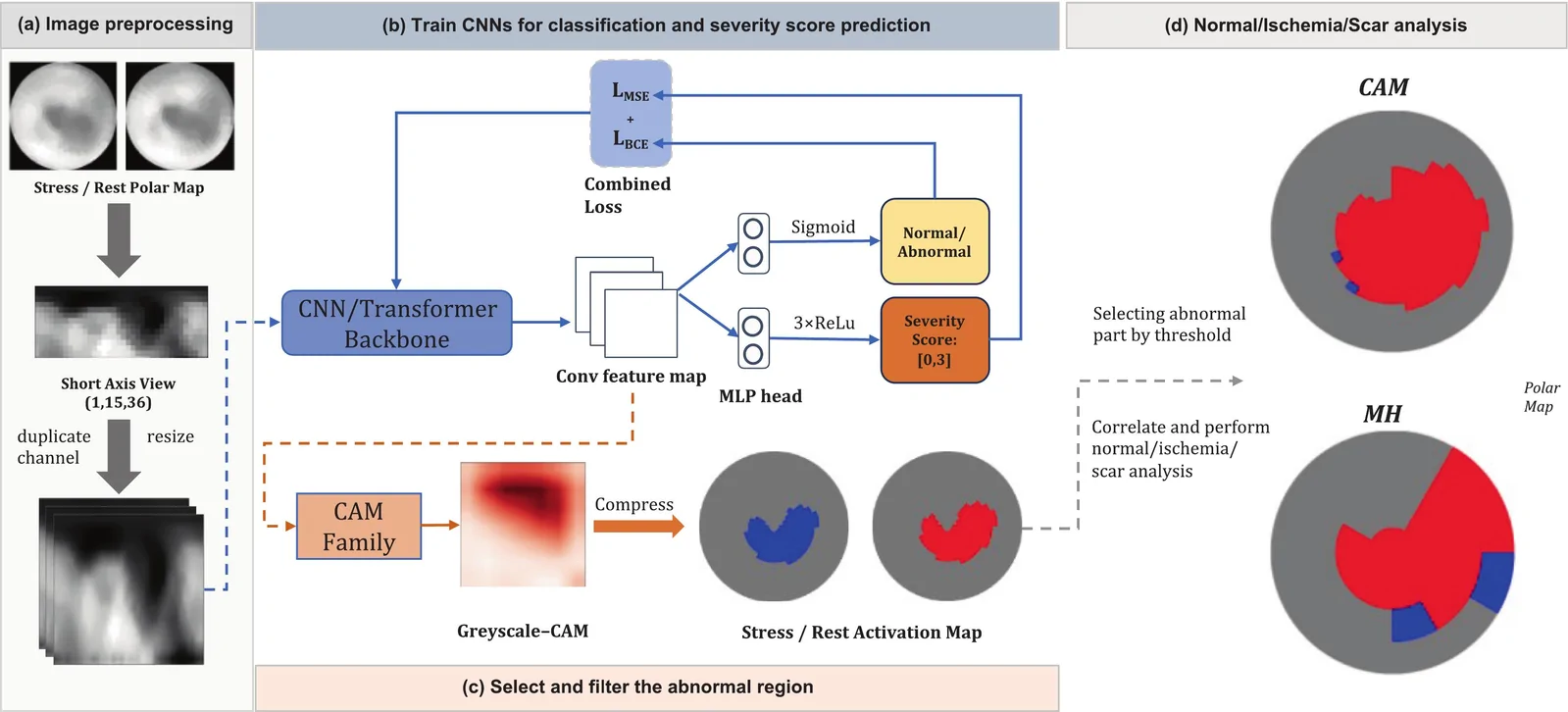
Step-by-step pipeline used in this project. a) Pre-processing: single polar maps (15,36) are adapted as ResNet inputs (3,224,224). b) Classification: the model is optimized with a two-target objective, $y_{i}$ and $s_{j}$. c) Localization: a CAM-based heatmap is used to find the most likely perfusion abnormality region. d) Post-processing: for both stress and rest maps, a threshold is set in concordance to the severity score prediction. The upper two images show abnormal region in stress polar map (left) and rest polar map (right). These abnormal regions in both polar maps are further combined with a normal-ischemia-scar (white/blue/red) analysis in the lower two images (left:CAM, right:TPD).

Our CNN implementation and pipeline are shown in Fig. 3. Since this model should output a diagnosis $y_{i}$ and a severity score $s_{j}$, we optimized it for both a classification and a regression task. The loss function is presented in $\left(4\right)$, including a binary cross entropy (BCE) loss for the classification, a mean squared error (MSE) loss for the severity estimation, and a regularization parameter:

(4) $$L_{\text{new}}\left(w\right)=\underset{\text{BCE loss}}{\underbrace{-\frac{1}{N}\sum_{i=1}^{N}\left[y_{i}\log\left(p_{i}\right)+\left(1-y_{i}\right)\log\left(1-p_{i}\right)\right]}}+\underset{\text{MSE Loss,}\;s_{i}\text{: severity}}{\underbrace{\alpha \cdot \frac{1}{N}\sum_{i=1}^{n}{\left(s_{i}-\hat{s_{i}}\right)}^{2}}}+\underset{\text{Weight Decay (L2-Penalty)}}{\underbrace{\lambda w^{T}w}}$$

As a severity measure we chose the Grad-CAM++ [29] applied on the last convolutional layer, which provides a localization map showing the positive influences of each voxel on the output. The selection of Grad-CAM++ over plain Grad-CAM [30] was mainly due to the possibility of finding more than one abnormal region in a SPECT polar map, in which case a weighted average of the gradients is expected to provide a better localization.

To determine the optimal architecture and to avoid data leakage, we conducted a preliminary model selection study on a representative subset of 221 studies of this data. Several candidate models were compared on a 60/20/20 split of this subset, including a simple CNN (with 2 layers), ResNet-18, ResNet-34, ResNet-50, U-Net segmentation networks [31] and YOLO-based object detection models [32]. The pilot results indicated that ResNet-18 [33] offered the optimal balance between performance and model complexity.

The subsequent training and evaluation in this work were performed on the full 1,028-patient cohort. The dataset was split into three subsets: 70% for training, 15% for validation and 15% for testing (720, 154 and 154 cases, respectively). We selected an SGD optimizer with learning rate 0.01, batch size of 64, OneCycleLR scheduler with 6000 total steps and patience of 150 steps for early stopping to avoid overfitting. The model was trained on a NVIDIA GeForce RTX 4080. The hyperparameter *α*, indicating the prevalence of the severity term in $\left(4\right)$, was optimized and set to 3 after an ablation study on the validation set.

To validate the results, there is no adequate ground truth. Instead, we compared our results with the conventional TPD to demonstrate their consistency. The comparison was first carried out visually on a patient-by-patient basis and the results shown through selected cases. The consistency between the two methods was then evaluated for the entire test cohort using various agreement metrics between the predictions of TPD and our new method (accuracy, precision, sensitivity, specificity, AUC). These metrics are presented for the overall localization agreement between the two methods and also for the agreement in each LV wall. Cross-modality comparisons (i.e. comparisons to coronary angiography stenosis assessed from cardiac CT images as golden standard) were not available for this cohort and are outside the scope of this work.

## Results

Preliminary Model Selection

On the small pilot subset of 221 studies, we compared a shallow 2-layer CNN with several residual backbones. As summarized in Table 1, all ResNet models outperformed the shallow CNN (agreement 85% vs. 74%). However, increasing depth did not monotonically improve performance. ResNet-34 showed identical agreement metrics to ResNet-18 but required nearly double the computational resources, whereas ResNet-50 yielded a slightly lower agreement (81%) and an extreme profile between sensitivity (25%) and specificity (100%). This pattern indicates overfitting of the deeper model to the majority normal class on our relatively small dataset.

**Table 1:** Preliminary comparison of CNN architectures on a small pilot subset. Metrics (agreement, sensitivity and specificity) are computed on a 60/20/20 split and are used solely to guide architecture selection; all main results in this work are based on the full 1028-patient cohort.

**Model**	**Agreement (%)**	**Sensitivity (%)**	**Specificity (%)**	**Trainable parameters**
**Simple CNN with 2 layers**	74	50	83	6421
**ResNet-18**	85	67	91	11180161
**ResNet-34**	85	67	91	21288321
**ResNet-50**	81	25	100	23513217

For completeness, we also ran preliminary experiments with U-Net segmentation networks and YOLO-style object detectors on the same 15×36 rectangularized polar maps. However, these architectures are conceptually mismatched to our setting: the available supervision is a global diagnostic/severity label rather than pixel-wise ground truth masks, and the 15×36 input resolution is much smaller than typical object-detection setups. In practice, the U-Net and YOLO models failed to achieve stable training and consistently showed inferior validation performance compared with the classification CNNs. We therefore do not report their detailed metrics in Table 1 and did not consider them further in the subsequent experiments on the full 1028-patient cohort. Consequently, ResNet-18 was selected as the backbone for the full-scale training.

Deep Learning Models Output

**Fig. 4:**
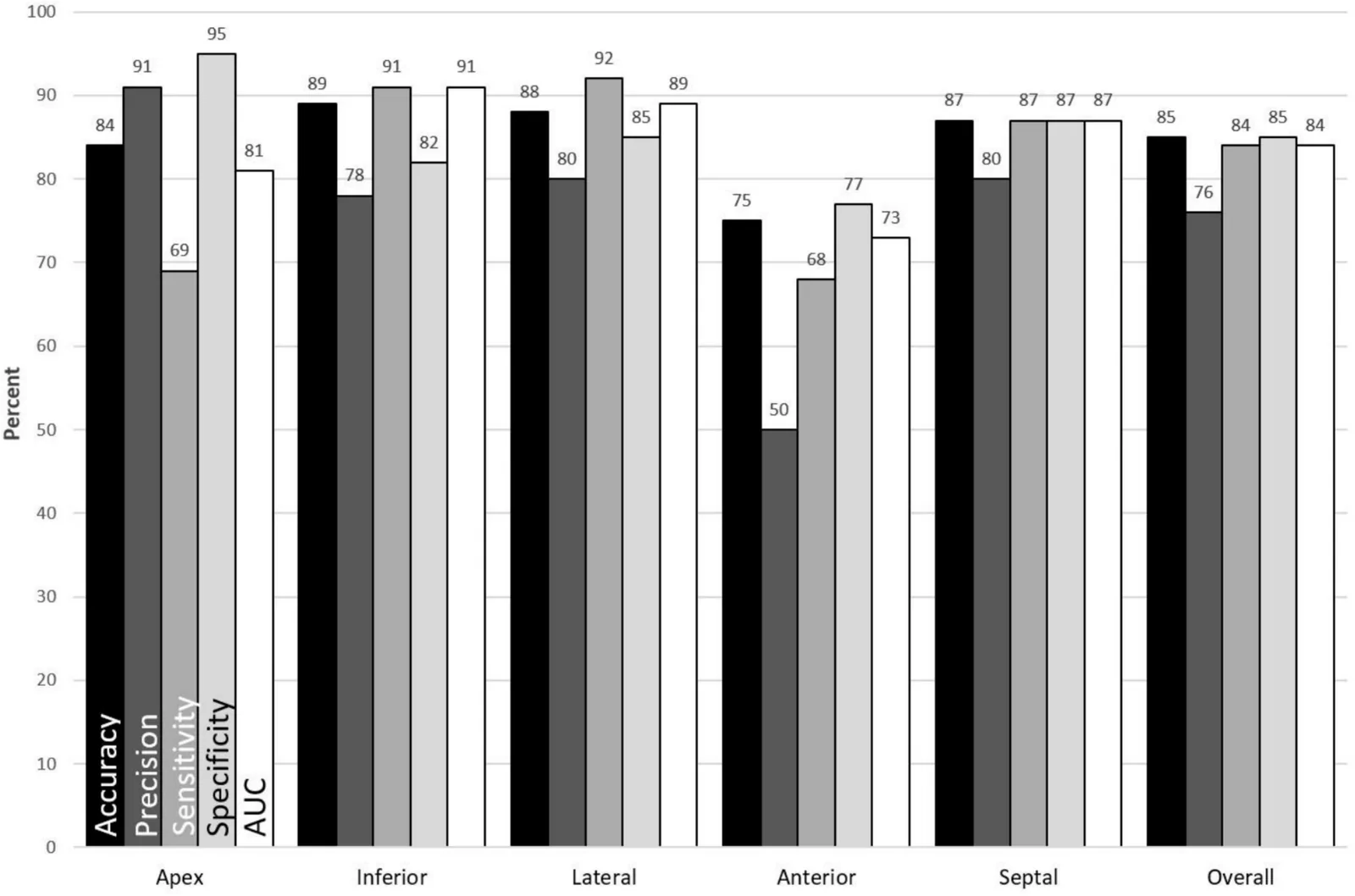
Localization consistency metrics between TPD and our method for each myocardial wall and overall. The abnormality of each myocardial wall in the polar map is defined as having at least one region (comprising more than 12 abnormal pixels in a specific myocardial wall region in MunichHeart) labeled as abnormal.

Fig. 4 shows the consistency metrics between CAM and TPD localization. We observe that both methods are generally consistent (overall localization accuracy above 85%) but they are also similarly consistent for every cardiac wall region (localization accuracy in the apical, inferior, lateral and septal regions above 80%). The predictions in the anterior wall were somewhat less consistent (75%).

**Fig. 5:**
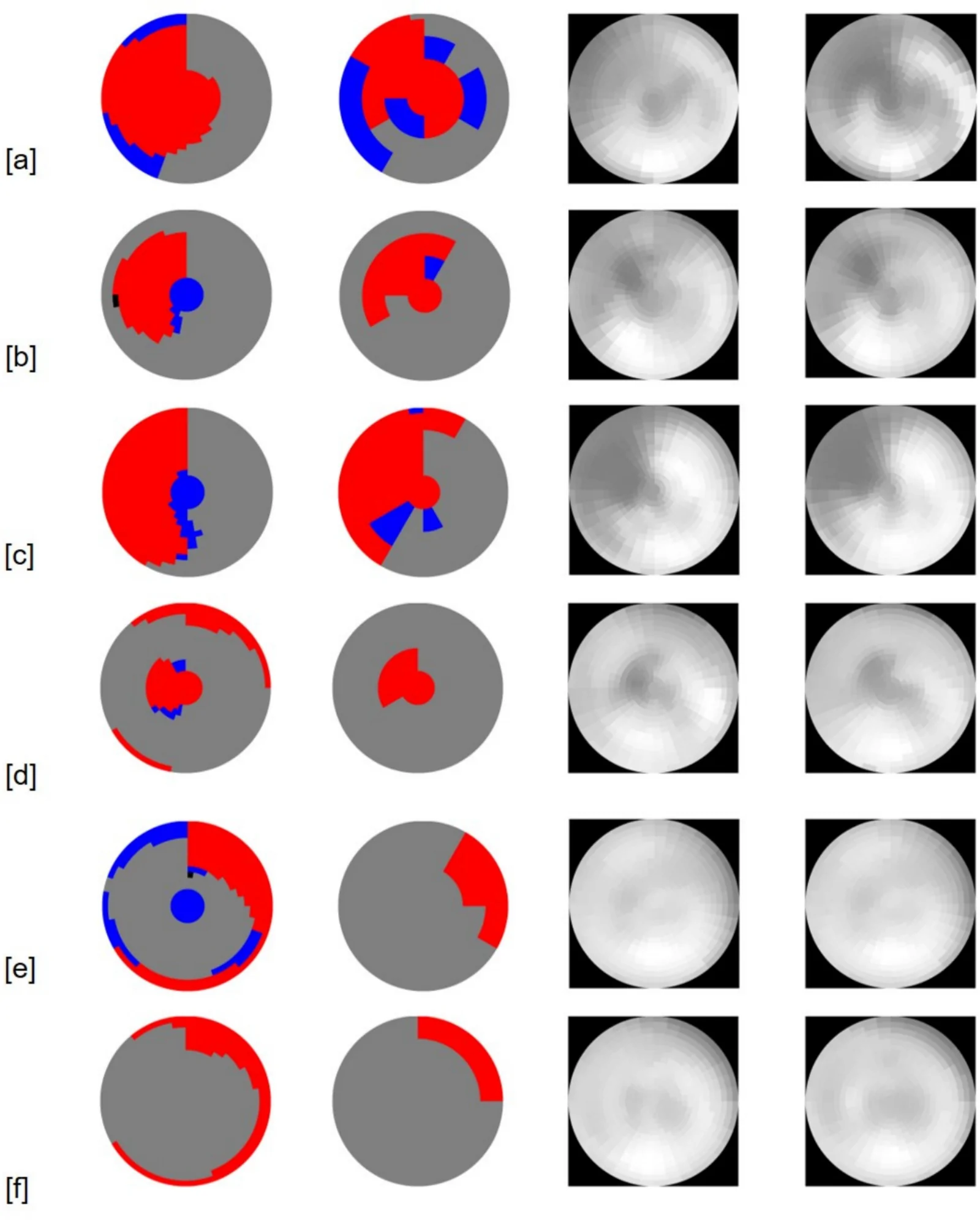
Localization of normal, ischemia, and scar tissue (n/i/s [%]: gray/blue/red) by our CAM-based approach (first column) and TPD (second column), and the corresponding stress/rest perfusion images (third and fourth column), with 6 patients. [a] male (77), TID: 80. n/i/s [%]: TPD 51/23/26, CAM 49/14/37 [b] male (64), TID: 109. n/i/s [%]: TPD 73/3/24, CAM 77/1/22 [c] male (72), TID: 119. n/i/s [%]: TPD 55/8/37, CAM 45/8/47 [d] male (62), TID: 105. n/i/s [%]: TPD 87/0/13, CAM 86/0/14 [e] female (63), TID: 76. n/i/s [%]: TPD 86/0/14, CAM 85/6/9 [f] female (80), TID: 112. n/i/s [%]: TPD 91/0/9, CAM 96/0/4

A visual comparison between our localization method and conventional TPD can be seen in Fig. 5. A general localization consistency between the two approaches can be visually assessed in the examples. One of the benefits of our method, namely the ability to perform localization at a voxel level, can also be observe.

**Fig. 6:**
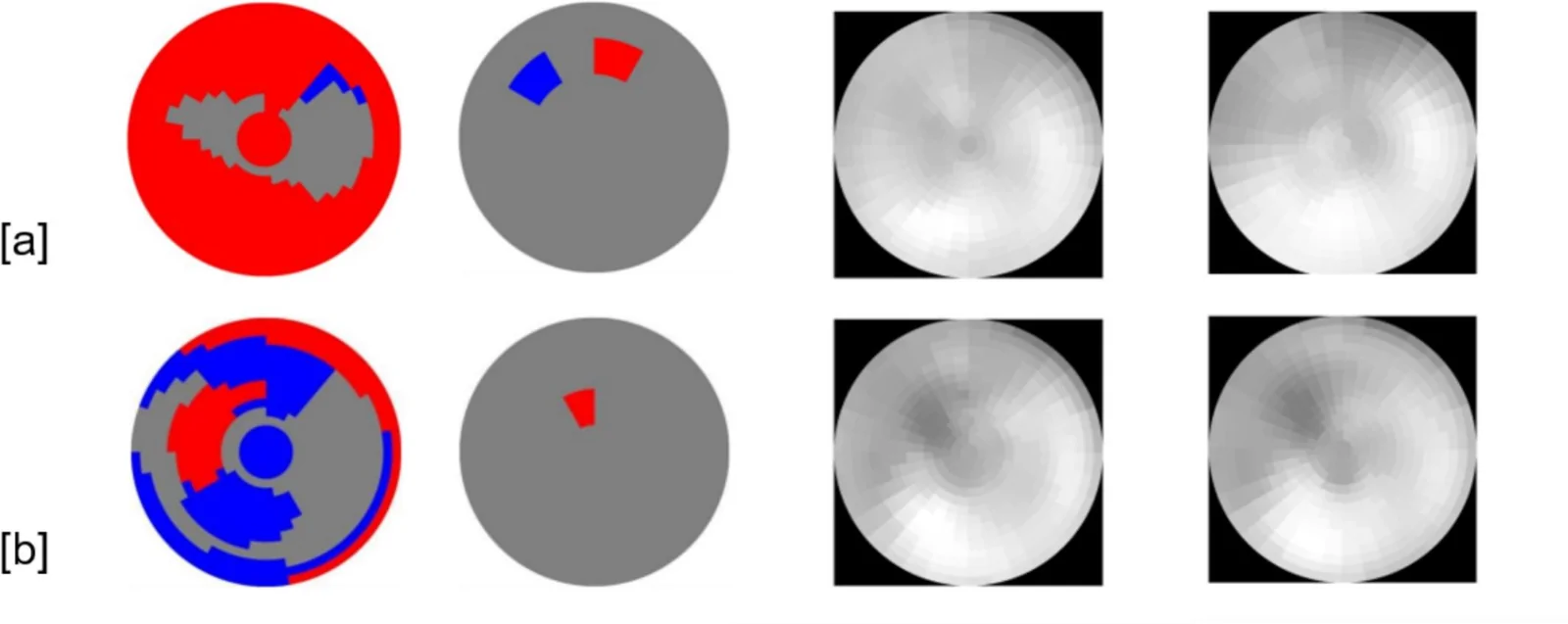
Examples of inconsistent localization between TPD (left) and CAM (right). [a] male (68), TID: 0.98. [b] female (61), TID: 1.42.

Around 9% [14] of the test cases resulted in a faulty or inconsistent localization with respect to TPD, as can be observed in Fig. 6.

The overall classification accuracy (normal vs. abnormal) of our method with respect to TPD is 80%. This is consistent with the overall localization accuracy (85%), showing that classification and localization are also coherent.

## Discussion

In this work, we presented a new approach to MPI quantification using SPECT. The mathematical analysis establishes the connection between our approach and TPD, indicating potential limitations of the TPD concept. Based on this foundation, an improved framework is proposed. A practical implementation is provided, demonstrating both visual and statistical consistency with the current paradigm. This new approach potentially offers an advantage over the conventional design as it provides the basis for including e.g. also non imaging parameters. Although this is a rather speculative statement, it reflects the authors experience that - in the end – the “clinical golden eye” is the reference on a decision on the per-patient decision. From this perspective, a new framework which performs clinically robust despite the mathematical complexity provides a methodological bridge between automated quantitative metrics and clinical expert judgment.

From the technical perspective - besides the expected training errors of the CNN - several factors contribute to the differences between the two methods:

1) our approach is inherently different from TPD;
2) TPD resulting from MunichHeart includes a user-dependent filtering process to eliminate smaller, non-significant parts of the localization before resampling the polar maps into lower resolution segments. Our method instead preserves the entire localization and does not resample into segments. Also, users usually filter out the mildly abnormal part in the normal-scar- ischemia analysis step;
3) severity score is calculated in a semi-quantitative way, so precision has been lost;
4) abnormal limit (threshold) is not investigated.

We tested deeper models (ResNet-34, 50) and transformer-based networks with a small pilot subset, but they were overfitted due to the small dataset (15x36 input size). ResNet-34 is the deepest model with reasonable results, but training performance does not surpass that of the more compact ResNet-18, which has substantially fewer trainable parameters than the deeper residual networks. Combined with standard regularization (early stopping based on validation performance), this reduced capacity makes severe overfitting less likely. While some degree of overfitting cannot be completely excluded given the dataset size, the pilot results in Table 1 indicate that the compact CNN achieves a more balanced trade-off between sensitivity and specificity and is less prone to overfitting than the deeper architectures. A well-designed, small network is needed in the next step. Both U-Net and YOLO-based architectures were also tested. However, U-Net is not well suited to this task because the available supervision is a global diagnostic/severity label rather than an anatomical segmentation mask, and the 15×36 rectangular representation does not correspond to a natural LV shape. YOLO-style object detectors, in turn, require bounding-box annotations and larger input images; given our small input size and weak labels, they did not converge to meaningful detections. Our initial trials worked without severity information but improved when using severity data.

**Fig. 7:**
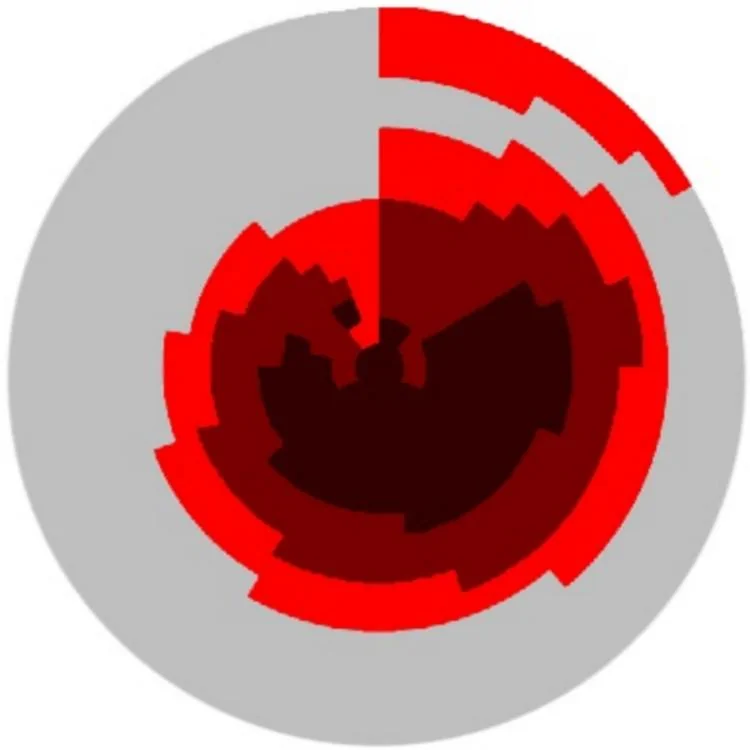
A semi-quantitative CAM segmentation of the abnormal region in a stress polar map (normal: gray; mild: red; moderate: deep red; severe: dark red). Male (64), TID: 1.07.

To facilitate further comparisons with TPD, our method permits the stepwise thresholding of the severity measure. This process generates a semi-quantitative severity score, analogous to the 4-point TPD score. Fig. 7 illustrates a potential segmentation of mildly, moderately, and severely abnormal regions, delineated by thresholds of 20%, 50%, and 80% of the measure *M*, respectively. Although it is technically possible to optimize these thresholds to best approximate the TPD scoring system, they serve primarily as a visual tool and have no real diagnostic value.

Our method was not based on per-segment statistics; however, it was able to consistently segment similar regions to TPD. This demonstrates that both methods identify comparable patterns to explain the deficit severity in the polar maps. However, as reported in the result, this was not consistently the case in a small set of cases. In some of these cases, the inconsistent segmentation between the two methods can be generally attributed to false positive cases resulting from an incomplete CNN training (i.e. overfitting caused by an incomprehensive training database, see cases [a] in Fig. 6). In other cases, nevertheless, there is a case that can be made for a false negative TPD localization. Case [b] in Fig. 6 may be an example of such a case, where a very high TID index value (1.42) and a low TPD indicate a three-vessel disease (3VD). The uniform myocardial ischemia throughout the entire LV due to combined stenosis of the three major coronary vessels in 3VD is a rare occurrence and usually yields false negative MPI results. High TID values, usually above 1.2 for both male and female subjects [34], have an 88% specificity for critical multivessel CAD [35] (up to 95% for TID values over 1.39 [36]), so a negative TPD diagnosis should be considered suspicious. In this case, our method shows high severity and extension of perfusion abnormality, but it remains to be studied if this new approach can consistently identify these rare occurrences.

It has been well studied that the TPD’s parameters $\theta \left\{\mu ,\sigma \right\}$ depend on population, scanner (Cadmium Zinc Telluride (CZT) or conventional), gender, radioisotope, stress type (exercise or pharmacologic stress) and patient position [7], [11]. Those factors will certainly also influence our new approach, and future models need to include either harmonization strategies or built-in priors $\left(f\left(X_{j}\right)+\lambda =s_{j}\right)$ to account for these effects. Another possibility is to develop site-specific protocols, similar to the normal-limit-based approach, and evaluate their impact in future studies.

In the theoretical section, it can be observed that our approach to localizing and quantifying perfusion defects can be categorized into two technical routes: solving a system of absolute value equations or using deep learning algorithms. The first route is suitable for small datasets, while the second is better suited for large datasets. For the first route $\left(Appendix\;B\right)$, pre-serving the first-order terms is equivalent to TPD. Higher-order terms are introduced as the training dataset size increases. Eliminating cross-correlation terms significantly reduces the number of parameters to be estimated, making this approach feasible for small datasets. The rationale behind this route is that not every site has access to many samples necessary to establish and apply neural networks. Additionally, the clinical community often prefers simple and robust approaches, which explains why TPD has remained prevalent for decades. In this paper, the actual implementation of the second route has been described. Further studies on this route will focus on optimizing the abnormal threshold limits, model design, and localization measures. Notably, the robustness of the neural networks is expected to be a key area of investigation in subsequent steps. The choice of a CNN with a ReLU-based model is motivated by its potential for convex relaxation, which offers analytical robustness certification for future studies.

In theory, our approach exhibits superior behavior compared to TPD due to the flexibility of parameterization within the function rather than relying solely on a simple Gaussian distribution. Simultaneously, using complex functions may introduce challenges such as reduced robustness and potential overfitting. However, these issues can be mitigated through meticulous model design and increasing the sample size. Another advantage of our quantification is its ability to account for nonlinear relationships between signal and tracer concentration. Unlike costly Positron Emission Tomography (PET) tracers like ^13^N ammonia or ^15^O radio water, which relatively adhere to a linear relationship and serve as the clinical gold standard for perfusion quantification, SPECT tracers exhibit relatively low extraction rates at high flows such as in stress [2], [37]. Traditional TPD assumes its linear relationship, whereas our approaches can incorporate it into their nonlinear functions. Furthermore, our framework and neural network design are not only well-suited for 2D SPECT-based perfusion imaging but also adaptable to 3D volumetric data, employing 3D CNNs for input. Moreover, our methodology can be extended to PET myocardial perfusion and MRI first-pass perfusion analysis. Many researchers have explored the prognostic value and risk stratification of the TPD approach [38], [39], [40]. Similarly, the prognostic power of our approach is supposed to be studied in the future.

Overall, the primary advantage of this framework is its modularity and flexibility. However, our study does have its limitations. One such limitation lies in the encoding of severity targets, which are derived from TPD results, thereby potentially influencing the encoded severity targets. While human labeling could be considered as an alternative, it lacks reproducibility across different experts. Additionally, further validation through multicenter data is necessary for clinical integration.

## Conclusion

In this paper, we presented a quantification method for myocardial perfusion imaging (MPI) enabled by a deep learning architecture. Within our framework, the current TPD paradigm can essentially be viewed as a linear regression with a max operator. While we have generalized this approach to a broader case, we presented and analyzed both consistent and inconsistent samples in the absence of log-term clinical ground truth. The quantification of MPI is particularly significant, as its value has been demonstrated in diagnosis, prognosis, and risk stratification. But analogous to TPD, which emerged 40 years ago, the exposure of our approach the clinical setting will be a challenge. Nevertheless, using such a machine-learning approach offers novel methodological perspectives in the interdisciplinary setting.

Credit authorship contribution statement

* Authors Yue Guo and Juncheng Ni share first co-authorship;

Conceptualization: Guo, Yue; Nekolla, Stephan G

Data Curation: Guo, Yue; Nekolla, Stephan G

Software: Guo, Yue

Formal analysis: Guo, Yue

Validation: Guo, Yue; Solari, Esteban Lucas; Ni, Juncheng

Methodology: Guo, Yue

Writing - Original Draft: Guo, Yue

Writing - Review & Editing: Guo, Yue; Solari, Esteban Lucas; Ni, Juncheng; Villagran Asiares, Alberto

Supervision: Nekolla, Stephan G

Resources: Nekolla, Stephan G

Disclosures and Funding

No.

## CRediT authorship contribution statement

**Yue Guo:** Writing – review & editing, Writing – original draft, Visualization, Validation, Software, Project administration, Methodology, Investigation, Formal analysis, Data curation, Conceptualization. **Juncheng Ni:** Writing – review & editing, Formal analysis. **Esteban Lucas Solari:** . **Alberto Villagran Asiares:** . **Stephan G. Nekolla:** Writing – review & editing, Supervision, Software, Funding acquisition, Data curation, Conceptualization.

## Declaration of competing interest

The authors declare that they have no known competing financial interests or personal relationships that could have appeared to influence the work reported in this paper.
